# Effect of Immunotherapy on Seizure Outcome in Patients with Autoimmune Encephalitis: A Prospective Observational Registry Study

**DOI:** 10.1371/journal.pone.0146455

**Published:** 2016-01-15

**Authors:** Jung-Ick Byun, Soon-Tae Lee, Keun-Hwa Jung, Jun-Sang Sunwoo, Jangsup Moon, Jung-Ah Lim, Doo Young Lee, Yong-Won Shin, Tae-Joon Kim, Keon-Joo Lee, Woo-Jin Lee, Han-Sang Lee, Jinsun Jun, Dong-Yub Kim, Man-Young Kim, Hyunjin Kim, Hyeon Jin Kim, Hong Il Suh, Yoojin Lee, Dong Wook Kim, Jin Ho Jeong, Woo Chan Choi, Dae Woong Bae, Jung-Won Shin, Daejong Jeon, Kyung-Il Park, Ki-Young Jung, Kon Chu, Sang Kun Lee

**Affiliations:** 1 Departments of Neurology, Seoul National University Hospital, Seoul, South Korea; 2 Program in Neuroscience, Seoul National University College of Medicine, Seoul, South Korea; 3 Department of Neurology, Ewha Womans University School of Medicine and Ewha Medical Research Institute, Seoul, South Korea; 4 Departments of Neurology, Samsung Medical Center, Seoul, South Korea; 5 Departments of Neurology, Chosun University Hospital, Gwangju, South Korea; 6 Departments of Neurology, Asan medical center, Seoul, South Korea; 7 Departments of Neurology, Ewha Woman’s University Hospital, Seoul, South Korea; 8 Departments of Neurology, Ajou University Medical Center, Suwon, South Korea; 9 Departments of Neurology, Konkuk University Medical Center, Seoul, South Korea; 10 Departments of Neurology, Inje University Busan Paik Hospital, Busan, South Korea; 11 Departments of Neurology, Kyungpook National University Hospital, Daegu, South Korea; 12 Departments of Neurology, St. Mary's Hospital, Seoul, South Korea; 13 Departments of Neurology, Cha university, CHA Bundang Medical Center, Seongnam, South Korea; University of Rome Tor Vergata, ITALY

## Abstract

**Objective:**

To evaluate the seizure characteristics and outcome after immunotherapy in adult patients with autoimmune encephalitis (AE) and new-onset seizure.

**Methods:**

Adult (age ≥18 years) patients with AE and new-onset seizure who underwent immunotherapy and were followed-up for at least 6 months were included. Seizure frequency was evaluated at 2–4 weeks and 6 months after the onset of the initial immunotherapy and was categorized as “seizure remission”, “> 50% seizure reduction”, or “no change” based on the degree of its decrease.

**Results:**

Forty-one AE patients who presented with new-onset seizure were analysed. At 2–4 weeks after the initial immunotherapy, 51.2% of the patients were seizure free, and 24.4% had significant seizure reduction. At 6 months, seizure remission was observed in 73.2% of the patients, although four patients died during hospitalization. Rituximab was used as a second-line immunotherapy in 12 patients who continued to have seizures despite the initial immunotherapy, and additional seizure remission was achieved in 66.6% of them. In particular, those who exhibited partial response to the initial immunotherapy had a better seizure outcome after rituximab, with low adverse events.

**Conclusion:**

AE frequently presented as seizure, but only 18.9% of the living patients suffered from seizure at 6 months after immunotherapy. Aggressive immunotherapy can improve seizure outcome in patients with AE.

## Introduction

Autoimmune encephalitis (AE) is an emerging cause of diffuse or limbic encephalitis that frequently presents with seizure or status epilepticus.[1, 2]. Neuronal antibodies of either paraneoplastic or nonparaneoplastic origin have been discovered to be associated with patients with autoimmune encephalitis.[3, 4] Paraneoplastic antibodies, including those against Hu, Ma2/Ta, amphiphysin, and CRMP5, involve the limbic system and cause seizures with memory deficit or psychiatric symptoms.[5] Nonparaneoplastic antibodies, including those against the anti-*N*-methyl-d-aspartate receptor (NMDAR), voltage-gated potassium channel (VGKC, leucine-rich glioma inactivated 1 (LGI1), or Caspr2), and gamma-aminobutyric acid b (GABAb), directly target synaptic proteins that play a critical role in synaptic transmission, which can cause seizures.[6, 7] Seventy percent of the patients with anti-NMDAR encephalitis develop complex partial seizure, generalized tonic–clonic seizure, or status epilepticus.[8, 9] The antibodies against LGI1 or GABAb are associated with classic limbic encephalitis and early prominent seizures.[6, 10] Notably, LGI1 encephalitis often presents with a characteristic seizure called faciobrachial dystonic seizure [11], which can precede the other symptoms of encephalitis.[12] Seizures can occur not only during the acute phase but also later, resulting in postencephalitic epilepsy because of frequent involvement of epileptogenic structures. Seizure itself can decrease quality of life and increase the risk of injuries and sudden unexpected death.[13] However, studies of seizure characteristics and outcome in AE are lacking.

Accumulating evidence supports the use of immunotherapies in AE to improve functional outcome. High-dose corticosteroids, intravenous immunoglobulin (IVIg), or plasma exchange are widely accepted as first-line immunotherapies; moreover, rituximab has been recommended recently as a second-line immunotherapy in patients for whom the first-line therapy has failed.[9, 14] However, the effect of immunotherapy on seizure outcome in AE has not been fully evaluated. One study reported on the long-term seizure outcome in patients with nonparaneoplastic limbic encephalitis; however, that study was limited by the small number of patients included.[15] Moreover, the study did not focus on the effect of immunotherapy on seizure outcome. Patients with autoimmune epilepsy responded to first-line immunotherapy in 62% of the cases; however, this result cannot be simply applied to patients with AE, as the proportion of antibody types differs greatly between patients with autoimmune epilepsy and those with AE.[16, 17]

In this study, we aimed to evaluate seizure characteristics and outcome after immunotherapy in patients with autoimmune encephalitis who presented with new-onset seizure.

## Materials and Methods

### Study participants and ethics

Patients with AE who visited, or whose samples were sent to, the Seoul National University Hospital were considered for inclusion in this study. Autoantibodies, including synaptic (anti-NMDAR, -LGI1, -CASPR2, -AMPA1, -AMPA2, and -GABAB-R) and onconeuronal (anti-Hu, -Yo, -Ri, -Ma2, -CV2/CRMP5, and -amphiphysin) antibodies, were tested using an indirect immunofluorescence test performed on serum or CSF (Euroimmune Ag, Germany), as described previously.[18, 19] The Clinical characteristic and outcome of the patient, and the result of diagnostic tests including brain magnetic resonance imaging (MRI), electroencephalogram (EEG), and cerebrospinal fluid (CSF) study were obtained from questionnaires filled out by study investigators or the referring physicians.

AE was defined using the following criteria [20]: (1) acute or subacute onset of symptoms of encephalitis, (2) evidence of CNS inflammation by either MRI or CSF analysis, (3) exclusion of other possible etiologies, and (4) positive serum or CSF neuronal antibody (either synaptic or onconeuronal). We included adult (age ≥ 18 years) patients with AE who developed new-onset seizure, underwent immunotherapy, and were followed-up for at least 6 months after the initiation of immunotherapy. Those with limited clinical information were excluded from the analysis. This study was approved by the Institutional Review Board of the Seoul National University Hospital. Written informed consent to participate was obtained from the patients enrolled or their next of kin.

### Clinical information

For each patient, demographics and clinical information, including seizure characteristics, use of antiepileptic drugs, and associated clinical symptoms (cognitive impairment, psychiatric symptoms, and movement disorders) were reviewed. The results of autoantibody tests, brain MRI, EEG, and CSF analysis, as well as the type of underlying neoplasm, were also recorded. SE was defined as prolonged or repetitive seizure without full gain of consciousness between episodes for more than 5 min.[21] We only evaluated convulsive SE in this study, as the differentiation of a nonconvulsive status from the disease process is often difficult in AE.[22] CSF leukocytosis was defined as a CSF WBC count > 5/mm^3^, and CSF protein elevation was defined as a CSF protein level that exceeded 35 mg/dL. Screening of neoplasms was performed by either chest and abdomen computed tomography or whole-body fluorideoxyglucose positron emission tomography, and only neoplasms that were diagnosed within 5 years of symptom onset were included in the study.

### Immunotherapy

The choice of the immunotherapy was based on the treating physician’s preference and acceptance of the treatment by the patient or his/her family. First-line immunotherapy was defined as the use of high-dose steroids, intravenous immunoglobulin, or plasma exchange, alone or combined.[9] High-dose steroids were administered at an equivalent dose of methylprednisolone of 500–1000 mg for 5 days, and IVIg was administered at dose of 0.4 g/kg for 5 days. Patients who continued to have seizures after the first-line immunotherapy received additional alternative first-line immunotherapy or second-line immunotherapy with rituximab. Rituximab was administered according to the following schedule: 375 mg/m^2^ of rituximab induction via weekly infusion for 4 weeks, and additional monthly administration of rituximab, if necessary.

### Outcome measures

The primary outcome measure was seizure frequency. We evaluated seizure frequency before immunotherapy and at 2–4 weeks and 6 months after the initial immunotherapy. Seizure frequency was categorized into three groups: Group 1, seizure remission; Group 2, > 50% significant seizure reduction; and Group 3, no change. The medical records of adverse events were reviewed and classified using the Common Terminology Criteria for Adverse Events (CTCAE v 4.0), as performed previously in this context.[23, 24] To measure functional status before and after the immunotherapy, modified Rankin scale (m RS) was recorded.

### Statistical analysis

We compared the demographics and clinical data according to autoantibody type, and evaluated seizure remission at 2–4 weeks and 6 months after immunotherapy initiation. Because the majority of patients had either anti-NMDAR or -VGKC antibodies, autoantibody type was categorized into the following three groups: anti-NMDAR, anti-VGKC (LGI1 or Capsr2), and others (anti-GABAb or onconeuronal antibody). Continuous data were compared using the Mann–Whitney *U* test, and Fisher’s exact test was used for the analysis of categorical data. Significance was set at *P* < 0.05. Data were expressed as median and range for continuous variables, and as counts (percentages) for categorical variables.

## Results

### Clinical features and demographics

From May 1, 2012, and July 1, 2014, 49 patients with AE presented with new-onset seizure. Eight patients were excluded from this study: six because of limited clinical information and two because they refused immunotherapy. None of the patients who refused immunotherapy had further clinical improvement. Finally, 41 AE patients who presented with new-onset seizure were included in this study. The median age at seizure onset was 43 years (range, 18–74 years), and 21 patients (51.2%) were male. Seizure had been present for a median of 29 days (range, 2–364 days) before immunotherapy. The neuronal antibodies detected were as follows: anti-NMDAR antibodies in 17 patients (41.5%), anti-VGKC complex antibodies in 17 patients (41.5%; 14 LGI1 and three Caspr2), anti-GABAb antibodies in three patients (7.31%; one patient had concomitant anti-Hu antibodies), and onconeuronal antibodies in four patients (9.75%; two with anti-Ma2/Ta, one with anti-Yo, and one with anti-amphiphysin antibodies). Twenty-one patients (nine with anti-NMDAR, six with anti-LGI1, three with anti-Caspr2, two with anti-GABAb, and one with anti-amphiphysin antibodies) were reported previously.[18, 19, 25–27] The details of the patients are listed in S1 Table.

Age at seizure onset was younger in patients with anti-NMDAR antibodies than it was in patients with other antibody types (median, 27 years vs 59 years for anti-VGKC and 66 years for other antibodies; *P* < 0.0001). At presentation, 12 (29.3%) patients had focal seizures without impaired awareness, 18 (43.0%) had focal seizures with impaired awareness, 21 (50.0%) had secondary bilateral convulsive seizures, and 11 (26.8%) had multiple seizure types. Seven patients (17.1%) presented with faciobrachial dystonic seizures (FBDS), and all patients had anti-LGI1 antibodies. Five patients (four with anti-NMDAR and one with anti-Ma2/Ta antibodies) had convulsive SE, and daily seizures occurred in 25 (61.0%) patients. With the exception of FBDS, which was a characteristic feature of anti-LGI1 encephalitis, seizure type and frequency were similar between antibody types.

Other clinical characteristics and ancillary test results were similar between antibody types, with the exception of CSF findings and underlying malignancy. Associated symptoms were cognitive impairment in 20 (48.8%) patients, psychotic symptoms in 24 (58.5%) patients, and movement disorder in 16 (39.0%) patients. The median modified Rankin scale was 3 (range, 1–5), and 10 (24.4%) patients had good functional outcome (mRS < 3) before immunotherapy. Brain MRI abnormalities was noted in 18 (43.9%) participants, and 35 (85.4%) had abnormal EEG. EEG epileptiform discharges were observed in 18 (43.9%) patients, and 22 (53.7%) patients had EEG slowing. Representative EEG findings in each antibody types are shown in S1 Fig. CSF leukocytosis was detected in 17 out of the 34 evaluated patients, with a median WBC count of 7 (range, 0–385). CSF protein elevation was detected in 17 (50.0%) patients, with a median CSF protein level of 47 (range, 19–235). More patients with anti-VGKC antibodies had a normal WBC count and protein level compared with those with other antibodies (*P*-value = 0.003 and 0.001, respectively). Underlying malignancy was screened in 37 patients and was detected in four patients: two patients with anti-GABAb antibodies had small cell lung cancer, one patient with anti-amphiphysin antibodies had non-small-cell lung cancer, and one patient with anti-Yo antibodies had meningioma). No patients with anti-NMDAR or -VGKC antibodies had underlying cancer in our study (Table 1).

**Table 1:** Clinical characteristics of the patients according to antibody type.

	Total	NMDAR	VGKC	Others	*P*-value
	n = 41	n = 17	n = 17	n = 7	
Sex (male)	21 (51.2)	7 (41.2)	9 (52.9)	5 (71.4)	0.396
Age at seizure onset (years)	43 (18–74)	27 (18–43)	59 (24–72)	66 (33–74)	< 0.0001
Seizure duration (days)	29 (2–364)	20 (5–364)	32 (2–273)	43 (4–120)	0.811
Seizure type					
FS–	12 (29.3)	4 (23.5)	6 (35.3)	2 (28.6)	0.752
FS+	18 (43.9)	9 (52.9)	9 (52.9)	0	0.037
GTCS	21 (51.2)	10 (58.8)	6 (35.3)	5 (71.4)	0.196
FBDS	9 (17.1)	0	9 (52.9)	0	< 0.0001
Multiple	11 (26.8)	6 (35.3)	5 (29.4)	0	0.197
SE	5 (25.0)	4 (23.5)	0	1 (14.3)	0.109
Daily seizure	25 (61.0)	10 (58.8)	12 (70.6)	3 (42.9)	0.436
Associated symptoms					
Cognitive	20 (48.8)	9 (52.9)	9 (52.9)	2 (28.6)	0.502
Psychiatric	24 (58.5)	13 (76.5)	8 (47.1)	3 (42.9)	0.143
mRS < 3 at adm	10 (24.4)	2 (11.8)	7 (41.2)	1 (14.3)	0.108
Abnormal MRI	18 (43.9)	5 (29.4)	10 (58.8)	3 (42.9)	0.224
EEG EDs	17 (41.5)	6 (35.3)	9 (52.9)	2 (28.6)	0.434
CSF study					
Leukocytosis	17/34 (50.0)	12 (75.0)	1 (9.1)	4 (57.1)	0.003
WBC count	7 (0–385)	16 (0–385)	0 (0–6)	7 (0–165)	0.365
Protein ↑	17/34 (50.0)	9 (56.3)	1 (9.1)	7 (100.0)	0.001
Protein level	47 (19–235)	38.8(20–102)	31.5 (19–55)	67 (39–235)	0.076
Underlying malignancy	4/37 (10.8)	0	0	4 (57.1)	< 0.0001

Abbreviations: NMDAR, *N*-methyl-d-aspartate receptor; VGKC, voltage-gated potassium channel; FS–, focal seizure without impairment of consciousness; FS+, focal seizure with impairment of consciousness; GTCS, generalized tonic–clonic seizure; FBDS, faciobrachial dystonic seizure; SE, status epilepticus; mRS, modified Rankin Scale; MRI, magnetic resonance imaging; EEG, electroencephalography; ED, epileptiform discharge; CSF, cerebrospinal fluid; WBC, white blood cells; Protein ↑, protein elevation

### Seizure outcome after immunotherapy

**Fig 1:**
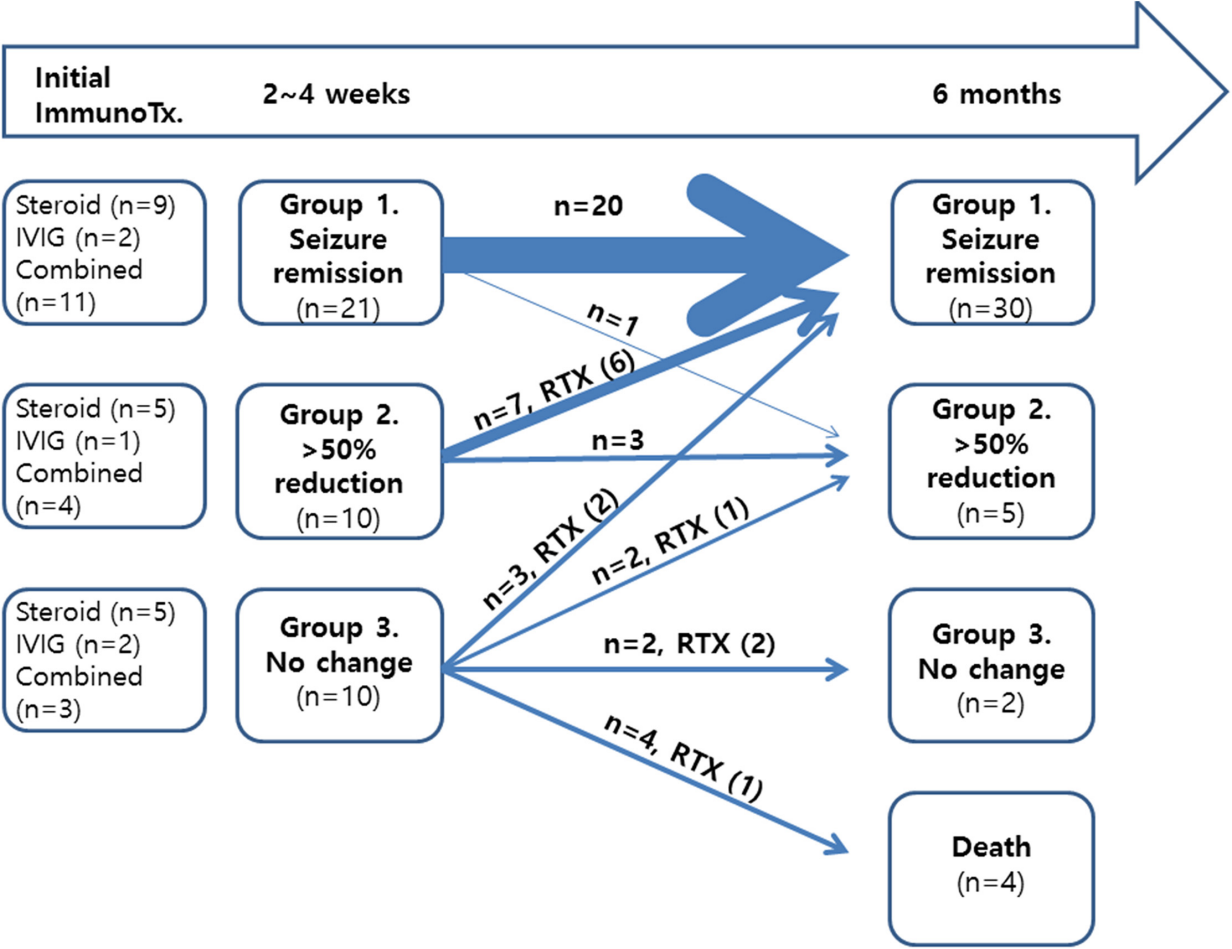
Flowchart of the response to immunotherapy at 2–4 weeks and 6 months after immunotherapy initiation. RTX (number of patients who received rituximab). Abbreviations: ImmunoTx, immunotherapy; RTX, rituximab.

As initial immunotherapy, 19 (46.3%) patients received high-dose steroids, five (12.2%) patients received IVIg, and 17 (41.5%) patients received both high-dose steroids and IVIg simultaneously. Two of the patients treated with high-dose steroid received plasma exchange as additional treatment. Seizure outcome after initial immunotherapy is displayed in Fig 1.

### Group 1: Seizure remission

At 2–4 weeks after the initial immunotherapy, 21 (51.2%) patients were seizure free. Three of these patients (two with anti-NMDAR and one with anti-GABAb antibodies with concomitant anti-Hu antibodies) entered seizure remission before immunotherapy with AED alone, and all remained seizure free at 6 months. One patient with anti-NMDAR encephalitis became seizure free after treatment with oxcarbazepine and topiramate, and two additional patients achieved that status after administration of levetiracetam alone. Eighteen patients with AED-refractory seizures (four with anti-NMDAR, 12 with anti-VGKC, one with anti-GABAb, and one with anti-Ma2/Ta antibodies) became seizure free only after the initial immunotherapy, and all except one patient remained seizure free at 6 months. One patient with LGI1 antibody encephalitis (PTS 21) initially presented with daily dialeptic seizure with olfactory aura, which completely stopped after receiving IVIG with high-dose steroids. She maintained seizure freedom with levetiracetam alone but relapsed 5 months after immunotherapy and had weekly seizures.

### Group 2: > 50% seizure reduction

Seizure frequency was significantly reduced in 10 patients (24.4%; six with anti-NMDAR, three with anti-VGKC, and one with anti-Yo antibodies) after the initial immunotherapy. Three of them received additional immunotherapy with IVIg or steroids but had no improvement. Rituximab was used as a second-line immunotherapy in six patients, who all became seizure free at 6 months. Only one patient with anti-NMDAR antibodies gained seizure remission without rituximab therapy, and no other patients had further seizure reduction at 6 months.

### Group 3: No change

Ten patients (24.4%; five with anti-NMDAR, two with anti-VGKC, and one with anti-GABAb, with anti-amphiphysin, and with anti-Ma2/Ta antibodies) had no changes in seizure frequency after the initial immunotherapy. Three of them were placed on alternative first-line immunotherapy, which had no significant effect. Six patients received rituximab, two of whom had seizure remission at 6 months and one of whom had significant seizure reduction. Only one patient with anti-NMDAR encephalitis (PTS 32), who developed daily focal seizures after SE, had seizure freedom without rituximab at 6 months after receiving high-dose steroids. Four patients died within 3 months of hospitalization, and no patient had seizure improvement. Three patients died of sepsis and one patient with NMDAR encephalitis (PTS 34) died of multiorgan failure due to uncontrolled status epilepticus despite rituximab therapy.

Overall, only seven living patients (18.9%; three with anti-NMDAR, two with anti-LGI1, one with anti-Yo, and one with anti-Ma2/Ta antibodies) suffered from seizure 6 months after the onset of the initial immunotherapy. Five of them had weekly seizures, and the remaining two suffered from daily seizures.

### Factors associated with seizure remission in AE

**Fig 2:**
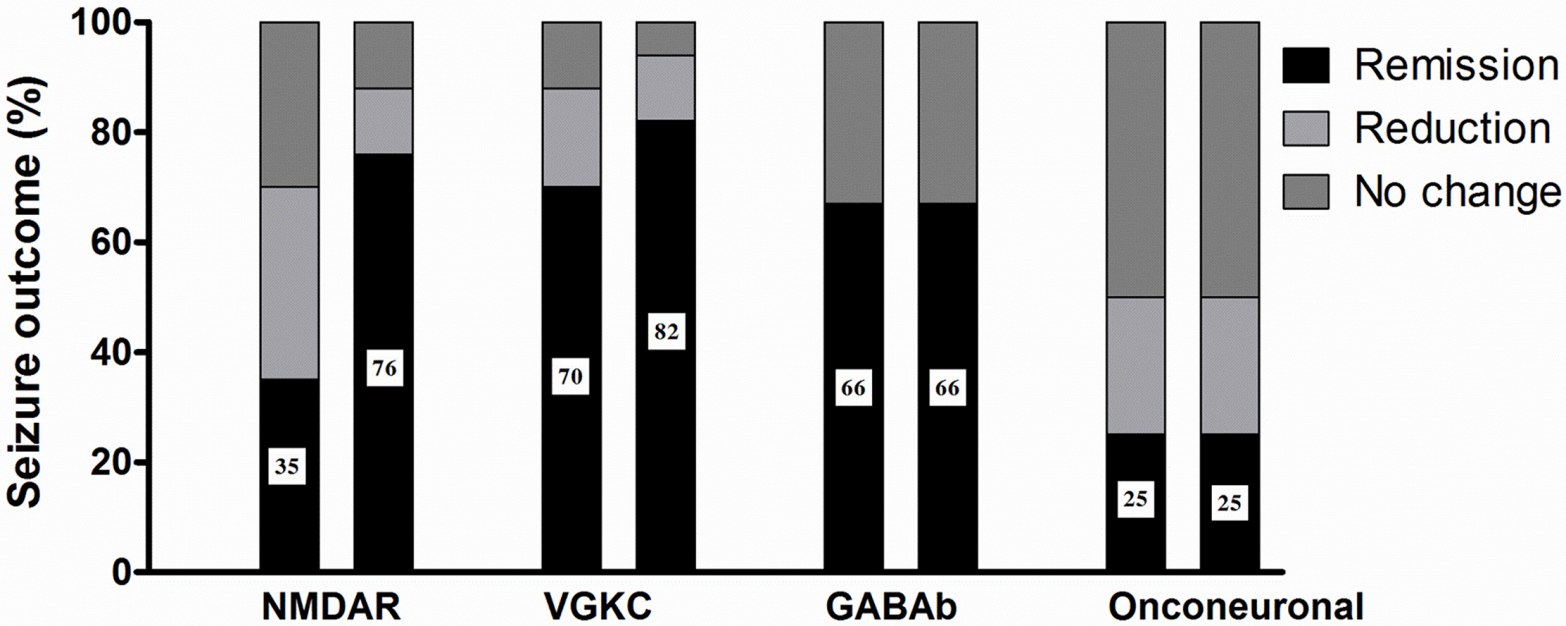
Seizure outcome at 2–4 weeks and 6 months after immunotherapy initiation according to underlying neuronal antibody. Left bar, seizure outcome at 2–4 weeks; right bar, seizure outcome at 6 months. Number: percentage of patients with seizure remission. Abbreviations: NMDAR, *N*-methyl-d-aspartate receptor; VGKC, voltage-gated potassium channel; GABAb, gamma aminobutyric acid b.

Overall, 21 (52.5%) patients had seizure remission at 2~4 weeks after initial immunotherapy. Immediate seizure response was similar between initial immunotherapy modalities (p = 0.685): steroid single (n = 19, seizure remission 47.4%, reduction 26.3%), IVIg single (n = 5, seizure remission 40%, reduction 20%), steroid with IVIg (n = 17, seizure remission 58.8%, reduction 17.6%). There was no difference in the seizure outcome according to autoantibody types (p = 0.107), however patients with VGKC encephalitis (12/17, 70.6%) tended to have more seizure remission after initial immunotherapy (*P* = 0.058, Fig 2). None of the five patients with SE had seizure remission after initial immunotherapy (*P* = 0.009). The median mRS after initial immunotherapy was 2 (range, 0–6), and 23 (56.1%) patients had good functional outcome (mRS < 3). More patients with seizure remission at 2~4 weeks also had good functional outcome (*P* < 0.001).

At 6 months after immunotherapy initiation, 30 (73.2%) patients were seizure free. However, only five patients were able to discontinue AEDs, and another 25 patients maintained a median of two AEDs. Those who continued to have seizures at 6 months were treated with additional AEDs during immunotherapy (median, four AEDs; *P* = 0.051) and had more AED escalation during immunotherapy. Patients with abnormal brain MRI (*P* = 0.036) or onconeuronal antibodies (*P* = 0.052) had poor seizure outcome at 6 months. All seven patients with FBDS (*P* = 0.083) were seizure free. Seizure outcome at 6 months was not associated with initial immunotherapy modality. The median mRS at 6 months was 2 (range, 0–6), and 27 (65.9%) patients had good functional outcome (mRS < 3). Those with seizure remission at 6 months also had better functional outcome (*P* = 0.016) (Table 2).

**Table 2:** Clinical characteristics of, and treatment administered to, patients with seizure remission or nonremission.

	Seizure remission 2–4wks	Nonremission 2–4 wks	*P*-value	Seizure remission 6 mo	Nonremission 6 mo	*P*-value
	n = 21	n = 20		n = 30	n = 11	
Sex (male)	11 (52.4)	10 (50.0)	1.000	14 (46.7)	7 (63.6)	0.484
Age at seizure onset (years)	54 (18–72)	38 (18–74)	0.251	51 (18–72)	42 (18–74)	0.768
Seizure duration (days)	25 (2–521)	33 (3–385)	0.735	24 (2–521)	37 (7–385)	0.458
FBDS	6 (28.6)	3 (15.0)	0.454	9 (30.0)	0	0.083
SE	0	5 (30.0)	0.021	3 (10.0)	2 (18.2)	0.598
Abnormal MRI	8 (38.1)	10 (50.0)	0.536	10 (33.3)	8 (72.7)	0.036
EEG EDs	8 (38.1)	9 (45.0)	0.756	12 (40.0)	5 (45.5)	1.000
CSF leukocytosis	4/14 (28.6)	13/20 (65.0)	0.080	10/24 (41.7)	7/10 (70.0)	0.259
CSF protein↑	9/14 (64.3)	8/20 (40.0)	0.296	11/24 (45.8)	6/10 (60.0)	0.708
mRS < 3 at adm	6 (28.6)	4 (20.0)	0.523	7 (23.3)	3 (27.3)	0.795
Follow-up mRS<3	17 (81.0)	6 (30.0)	0.001	23 (76.7)	4 (36.4)	0.016
Neural Ab type						
NMDAR	11 (55.0)	6 (28.6)	0.118	13 (43.3)	4 (36.4)	0.736
VGKC	12 (57.1)	5 (25.0)	0.058	14 (46.7)	3 (27.3)	0.309
GABAb	2 (9.5)	1 (5.0)	1.000	2 (6.7)	1 (9.1)	1.000
Onconeuronal	1 (4.8)	3 (15.0)	0.343	1 (3.3)	3 (27.3)	0.052
Underlying neoplasm	2/18 (11.1)	2/19 (10.5)	1.000	2/26 (7.7)	2/11 (18.2)	0.567
AED						
No. before ITx.	2 (1–5)	3 (1–4)	0.016	2 (1–5)	3 (1–4)	0.085
Maximum No.	N/A	N/A		2 (1–5)	4 (1–6)	0.051
AED escalation	N/A	N/A		9 (30.0)	7 (63.6)	0.074
Immunotherapy						
Initial steroid	9 (42.9)	10 (50.0)	0.758	15 (50.0)	4 (36.4)	0.499
Initial IVIg	2 (9.5)	3 (15.0)	0.663	3 (10.0)	2 (18.2)	0.598
Initial Steroid+IVIg	10 (47.6)	7 (35.0)	0.530	12 (40.0)	5 (45.5)	1.000

Abbreviations: wks, weeks; mo, months; FBDS, faciobrachial dystonic seizure; SE, status epilepticus; MRI, magnetic resonance imaging; EEG, electroencephalography; ED, epileptiform discharge; CSF, cerebrospinal fluid; mRS, modified Rankin Scale; WBC, white blood cells; Protein↑, protein elevation; NMDAR, *N*-methyl-d-aspartate receptor; VGKC, voltage-gated potassium channel; GABAb, gamma aminobutyric acid b; AED, antiepileptic drug; No., number; ITx, immunotherapy; IVIg, intravenous immunoglobulin

### Effect of rituximab on seizure outcome

Twelve among the 20 patients who continued to have seizures after the initial immunotherapy received rituximab as a second-line immunotherapy. Four patients received additional steroid or IVIg therapy before rituximab but had no seizure improvement. The details of these patients are listed in Table 3. Eight patients had anti-NMDAR antibodies, three had anti-LGI1 antibodies, and only one patient with anti-Ma2/Ta antibodies received rituximab treatment. The patients who were administered rituximab treatment received immunotherapy earlier than those who were not (median, 15.5 vs 75.5 days; *P* = 0.001) but had similar clinical characteristics and ancillary test results (S2 Table).

**Table 3:** Summary of the patients who received rituximab as a second-line immunotherapy.

Sex/Age	Ab type	ImmunoTx. (Additional Tx.)	Seizure outcome at 2–4 weeks (mRS)	From initial ITx. to RTX (wks)	RTX cycle	Seizure outcome at 6 months (mRS)	Side effect
F/18	NMDAR	Steroid+IVIg	RD (5)	3	#8	RM (4)	None
F/24	NMDAR	IVIg (Steroid)	RD (5)	4	#4	RM (5)	None
F/37	NMDAR	Steroid	RD (5)	3	#4	RM (2)	None
M/31	NMDAR	Steroid+IVIg	RD (4)	7	#1	RM (2)	Skin rash, fever for 1 day after
M/18	NMDAR	Steroid(IVIg)	NC (5)	3	#4	RM (4)	None
M/27	NMDAR	Steroid+IVIg	NC (4)	9	#8	RD (4)	None
M/43	NMDAR	IVIg	NC (5)	2	#5	NC (5)	None
F/26	NMDAR	Steroid+IVIg	NC (5)	2	#2	NC (6)	None, deceased*
F/42	LGI1	Steroid(IVIg, Plasmapheresis)	RD (3)	2	#7	RM (2)	None
F/68	LGI2	Steroid+IVIg	RD (2)	2	#6	RM (0)	Chest discomfort, headache during infusion
M/57	LGI1	Steroid (IVIg)	NC (2)	2	#4	RM (0)	Mild dizziness, headache during infusion
M/33	Ma2/Ta	IVIg	NC (2)	2	#6	NC (2)	None

Abbreviations: ITx, immunotherapy; RTX, rituximab; M, male; F, female; NMDAR, *N*-methyl-d-aspartate receptor; LGI1, leucine-rich glioma inactivated 1; IVIg, intravenous immunoglobulin; RM, remission; RD, reduction (> 50%); NC, no change

Eight out of the 12 (66.6%) patients who received rituximab gained seizure freedom at 6 months, and one patient had significant seizure reduction after rituximab therapy. However, two patients (one with anti-NMDAR and one with anti-Ma2/Ta antibodies) had no seizure improvement despite rituximab therapy. Compared with the eight patients who received no further immunotherapy, those who received rituximab had no significant improvement in seizure outcome (seizure remission, 66.6% vs 25.0%, *P* = 0.170; seizure reduction, 75.0% vs 25.0%, *P* = 0.065). However, among those who had significant seizure reduction after the initial immunotherapy, rituximab therapy was associated with more seizure remission at 6 months (100% vs 25%, *P* = 0.033).

### Adverse events

Three patients complained of adverse side effects from rituximab treatment. All adverse events were grade one or two (mild to moderate) in severity [23]: two patients had infusion-related reactions (mild headache, dizziness, and chest discomfort), whereas one patient had rash with pruritus 24 h after the infusion. Only one patient with pruritus had to discontinue treatment. In the remaining patients, the adverse symptoms subsided after reducing the infusion rate. One patient with anti-NMDAR antibody encephalitis (PTS 38) died after 2 weekly cycles of rituximab. The cause of death was uncontrolled status epilepticus and rigidity, which resulted in rhabdomyolysis and subsequent multiorgan failure. Rituximab was not directly associated with the cause of death in this patient.

## Discussion

In patients with autoimmune encephalitis and new-onset seizures, we observed dramatic seizure improvement after immunotherapy. At 6 months after immunotherapy initiation, 73.2% of the patients were seizure free, and 12.2% had significant seizure reduction. Because most of the patients were refractory to a median of two antiepileptic drugs, this result highlights the importance of immunotherapy in treating autoimmune-mediated epilepsy. A more aggressive immunotherapy with rituximab yielded additional seizure remission in 66.6% of the patients, especially in those who had partial response to the initial immunotherapy.

The percentage of seizure remission after immunotherapy in our study was higher than that of previous studies.[15, 16] In 14 patients with nonparaneoplastic AE and new-onset seizure, only 50% of the patients were seizure free after a mean 3-year follow-up from symptom onset.[15] In patients with presumed autoimmune epilepsy who presented with exclusive or predominant seizures, seizure improvement was reported in 62% of the cohort after intravenous delivery of methylprednisolone or IVIg, and only half of them had seizure remission.[16] The exclusive inclusion of patients with positive neuronal antibodies might account for the more favorable response obtained in our patients, as the previous literature indicated that patients with autoantibodies respond better to immunotherapy than do those without them.[16, 17] In addition, our patients received early immunotherapy (within 29 days from seizure onset). Compared with a previous study (a median of 10 months in responders and 22 months in nonresponders) [16], immunotherapy was offered much earlier in our patients. Early immunotherapy is well known to induce a better response.[9, 16, 17] However, the anti-GAD65 antibody, which is often associated with immunotherapy-refractory epilepsy (19), was not assessed in our patients, which could have resulted in a better response to the immunotherapy.

Despite the promising seizure-improvement effect of rituximab, this result of our study was not statistically significant. However, in the subgroup of patients who showed partial response to first-line immunotherapy, rituximab significantly reduced seizures compared with those who had no further immunotherapy. Rituximab is a monoclonal antibody directed against CD20, which induces B-cell depletion.[28] Partial response to first-line immunotherapy may imply an active immune-mediated disease process, and rituximab may have enforced this immune modulation. A previous study highlighted the benefits of the use of alternative immunosuppressants in initial nonresponders.[16] However, in our study, six patients who were placed on alternative first-line immunotherapy after failure of the initial treatment showed no improvement in seizure frequency. We suggest that patients who continue to have seizures after the initial immunotherapy should be placed on rituximab treatment rather than trying other first-line agents.

Patients with anti-VGKC antibodies tended to have more seizure remission after the initial immunotherapy. FBDS occurred only in patients with anti-LGI1 antibodies, who responded well to immunotherapy, especially to corticosteroids, as reported previously [11, 12, 29]. In contrast to a previous study that reported a questionable effect of rituximab in LGI1 encephalitis [6], all three patients with FBDS who received rituximab had additional seizure remission in our study. This result suggests a potential benefit of rituximab in patients with LGI1 encephalitis and FBDS. With the exception of those with FBDS, no patients with anti-VGKC antibodies showed further seizure improvement after the initial immunotherapy.

Conversely, patients with anti-NMDAR encephalitis initially had poorer seizure response to the initial immunotherapy. Only 35.3% of the patients had seizure remission within 1 month after the initial therapy. However, an additional 41.2% of the patients became seizure free within 6 months. As rituximab was started in eight out of 11 patients with anti-NMDAR encephalitis for whom the initial immunotherapy failed, this seizure reduction may be attributed to rituximab. However, there was no significant association between rituximab administration and seizure outcome (*P* = 0.528). Patients with anti-NMDAR encephalitis respond slowly to immunotherapy [1], and the recovery can take many months.[9] Moreover, in anti-NMDAR encephalitis, the frequency and intensity of seizure decrease as the disease progresses.[22] Accordingly, two of the patients who showed partial or no response after the initial immunotherapy became seizure free at 6 months, without receiving additional immunotherapy. Seizure improvement in anti-NMDAR encephalitis may not be dramatic right after the initial immunotherapy; however, the majority of patients (76.5%) eventually had seizure remission.

Five of our patients exhibited convulsive status epilepticus. With the exception of one patient with anti-Ma2/Ta antibodies, all patients had anti-NMDAR antibodies. The anti-NMDAR antibody is well known to be associated with status epilepticus.[30] Six refractory cases of status epileptics were reported among 100 patients with anti-NMDAR encephalitis.[8] Moreover, the anti-NMDAR antibody was detected in six among 31 patients with encephalitis and refractory SE who were admitted to an intensive care unit.[31] As the binding of autoantibodies to ionotropic receptors can change physiological cortical electric activities, autoimmune SE is often refractory to antiepileptic treatment and only responds to immunotherapy.[32] In three of our patients, SE resolved only after immunotherapy; however, these patients later developed intermittent refractory seizures after cessation of SE, which improved after rituximab therapy. It is important to know that AE patients with SE can later develop intractable seizures, which can be managed with aggressive immunotherapy.

The interpretation of the results of this study should be carried out in light of its limitations. This was a retrospective study that included a small number of patients. However, to date, this was the largest study of AE patients with seizure. Because not all patients underwent CSF antibody screening, there was a risk of false-positive or -negative results on the neuronal antibody test. Also detailed cognitive outcome and EEG improvement was not fully evaluated. Four patients with onconeuronal antibodies tended to show poor response to immunotherapy, which was in line with a previous report.[33] However, because of the small number of patients, the effect of immunotherapy on seizure outcome cannot be elucidated in these patients, and this calls for further studies. Moreover, rituximab was tried in selected patients with anti-NMDAR or -LGI1 antibodies, and in those who received early initial immunotherapy. Only one patient with an onconeuronal antibody (anti-Ma2/Ta) received rituximab and exhibited no significant improvement. Therefore, the benefit of rituximab observed in our study cannot be simply extended to all AE patients with other antibodies.

## Conclusions

Autoimmune encephalitis patients who presented with new-onset seizures exhibited dramatic seizure improvement after immunotherapy. Moreover, second-line immunotherapy with rituximab yielded further seizure reduction with a relatively low level of adverse events in patients who had partial response to the initial immunotherapy. These findings support the implementation of more aggressive immunotherapy to improve seizure outcome in patients with antibody-positive autoimmune encephalitis. Additional prospective trials with a longer follow-up period will be necessary to confirm these findings.

## Supporting Information

S1 Fig Representative EEG findings before and after immunotherapy in each antibody types. NMDAR: Eighteen year-old female with anti-NMDAR encephalitis initially had continuous rhythmic delta activity, bi-frontal dominant. Six months after immunotherapy, her follow-up EEG only showed mild diffuse slow waves. VGKC: Sixty eight year-old female with anti-LGI1 encephalitis initially had periodic lateralized epileptiform discharges on the left temporal areas. Her follow-up EEG was normal at 6 months after immunotherapy. Others: Thirty-three year-old male with anti-Ma2 encephalitis initially had diffuse theta to delta slow waves. Six months after immunotherapy, follow-up EEG was normal except for excessive beta activity due to use of benzodiazepine.
(TIF)

S1 Table Detailed clinical characteristics and response to immunotherapy of individual patients. (DOC)

S2 Table Characteristics of patients with failure of the initial immunotherapy with or without rituximab. (DOC)
