# Supplementary figures and images for: Effect of Immunotherapy on Seizure Outcome in Patients with Autoimmune Encephalitis: A Prospective Observational Registry Study
Source: PLoS One. 2016 Jan 15;11(1):e0146455. doi: 10.1371/journal.pone.0146455 (PMC4714908; doi:10.1371/journal.pone.0146455)

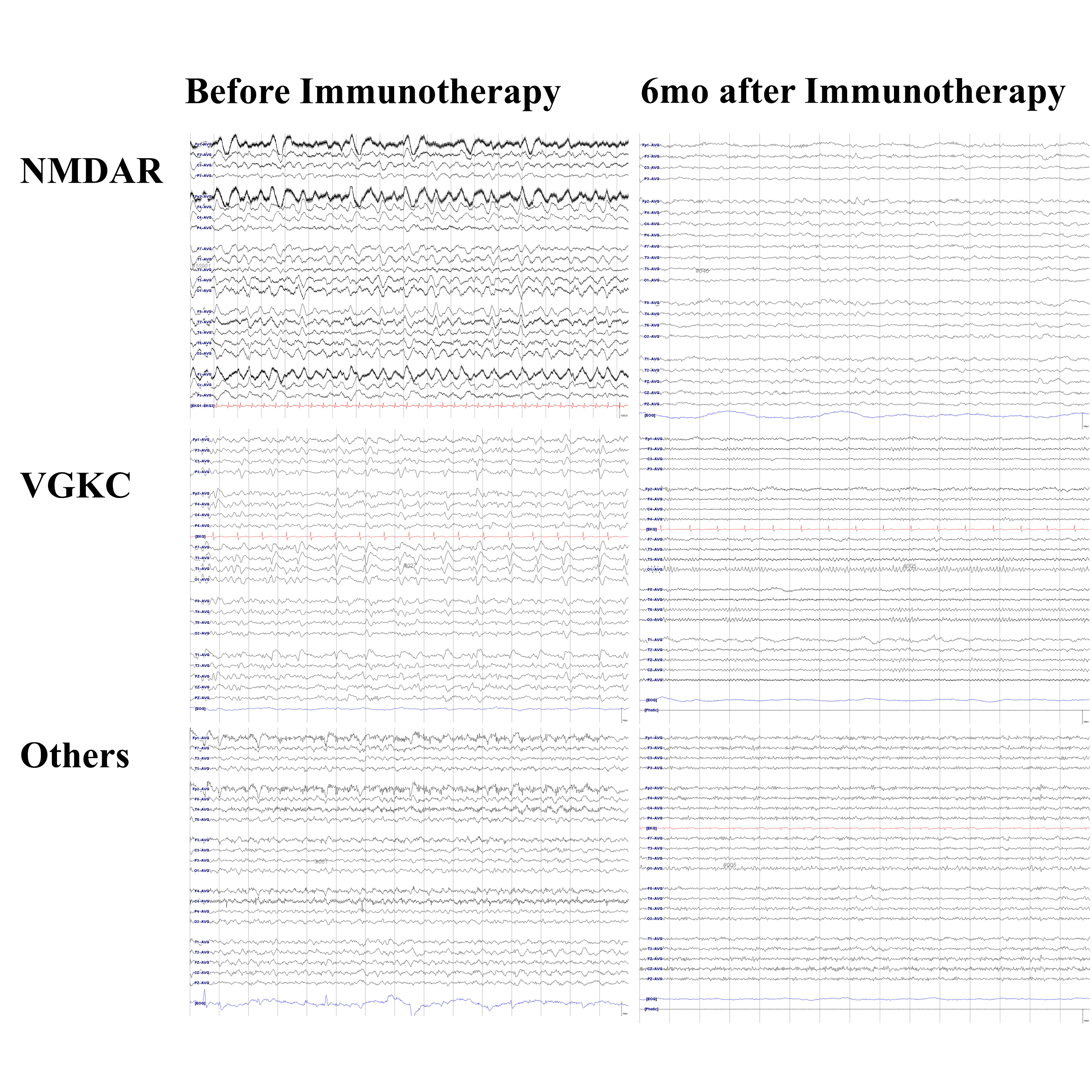

Supplement: S1 Fig — NMDAR: Eighteen year-old female with anti-NMDAR encephalitis initially had continuous rhythmic delta activity, bi-frontal dominant. Six months after immunotherapy, her follow-up EEG only showed mild diffuse slow waves. VGKC: Sixty eight year-old female with anti-LGI1 encephalitis initially had periodic lateralized epileptiform discharges on the left temporal areas. Her follow-up EEG was normal at 6 months after immunotherapy. Others: Thirty-three year-old male with anti-Ma2 encephalitis initially had diffuse theta to delta slow waves. Six months after immunotherapy, follow-up EEG was normal except for excessive beta activity due to use of benzodiazepine. (TIF) [file pone.0146455.s001.tif]
