# Supplementary material for: Effect of Immunotherapy on Seizure Outcome in Patients with Autoimmune Encephalitis: A Prospective Observational Registry Study
Source: PLoS One. 2016 Jan 15;11(1):e0146455. doi: 10.1371/journal.pone.0146455 (PMC4714908; doi:10.1371/journal.pone.0146455)
Supplement: S1 Table — (DOC) [file pone.0146455.s002.doc]

| S1 Table. Detailed clinical characteristics and response to immunotherapy of individual patients. | | | | | | | | | | | | |
| --- | --- | --- | --- | --- | --- | --- | --- | --- | --- | --- | --- | --- |
| Patient No/Sex/Age | Epilepsy duration (days) | Seizure type/  Frequency | EEG abnormality | Brain MRI | CSF WBC  /Protein | AutoAb type | Accompanying Sx | Initial immunoTx (initial AED) | Response at 2–4weeks | Additional immunoTx.  (maintenance) | Response at 6 mo. (AED use at 6 mo.) | mRS before immunotherapy → at 2–4 weeks → at 6 months  (underlying malignancy) |
| 1/M/38* | 324 | GTCS/Twice, FS+/Twice | RS | Normal | 5/53 | NMDAR | C, P | IVIG  (OxCBZ, TPM, PGB) | Remission | None | Remission (OxCBZ) | 3→2→1 |
| 2/F/59¶ | 2 | GTCS/Twice, FS/Daily | Normal | Normal | 0/55 | VGKC (LGI1) | p | Steroid  (LEV) | Remission | None  (Oral Pd) | Remission  (LEV) | 3→1→1 |
| 3/M/72¶ | 273 | FBDS/Weekly | IED | Normal | N/A | VGKC (LGI1) | C, M | IVIG  +Steroid  (LEV, CZP) | Remission | None | Remission  (LEV, TPM) | 2→2→0 |
| 4/F/18* | 17 | GTCS/3times, FS/Once | RS | Normal | N/A | NMDAR | C, P | Steroid  (VPA, DPH) | Remission | None  (Oral Pd) | Remission (None) | 4→3→3 |
| 5/M/57 | 120 | FS/Weekly | Normal | Normal | 0/53 | Ma2/Ta | C | IVIG  +Steroid  (OxCBZ, LEV) | Remission | None | Remission (OxCBZ, LEV) | 3→1→1 |
| 6/M/24° | 3 | GTCS/Daily | GS | Normal | 6/28 | VGKC (Caspr2) | P | IVIG  +Steroid  (LEV, VPA, TPM, DPH, CBZ) | Remission | None | Remission (LEV, VPA, TPM, DPH, CBZ) | 4→1→1 |
| 7/M/49° | 246 | FS/Daily, GTCS/Twice | RS | Normal | N/A | VGKC (Caspr2) | – | IVIG  +Steroid  (OxCBZ, LEV) | Remission | None  (Oral Pd) | Remission (LCM) | 2→1→1 |
| 8/F/64° | 40 | FS/ Weekly | RS | Bilateral medT T2 HSI | 0/22 | VGKC (Caspr2) | – | Steroid  (LEV) | Remission | None | Remission  (LEV) | 1→0→0 |
| 9/F/61¶ | 39 | GTCS/Weekly | IED | Bilateral medT T2 | 0/38 | VGKC (LGI1) | C | Steroid  (unknown) | Remission | None | Remission (unknown) | 2→1→1 |
| 10/M/59¶ | 32 | FBDS/Daily | RS | Normal | 0/35 | VGKC (LGI1) | C, P, M | IVIG  +Steroid  (LEV) | Remission | None  (Oral Pd) | Remission (OxCBZ) | 3→2→1 |
| 11/F/61 | 25 | FBDS/Daily | RS | Normal | N/A | VGKC (LGI1) | C, M | IVIG  +Steroid  (LEV, TPM) | Remission | None | Remission  (LEV, TPM) | 2→1→1 |
| 12/M/55¶ | 23 | GTCS/Once, FBDS/Daily | RS | Lt. medT T2 HSI | 0/35 | VGKC (LGI1) | P, M | IVIG  +Oral Pd (Oral Pd) | Remission | None  (Oral Pd) | Remission  (VPA, LTG) | 3→2→1 |
| 13/M/65¶ | 14 | FBDS/Daily | Normal | Normal | N/A | VGKC (LGI1) | M | Steroid  (DPH, CZP) | Remission | None  (Oral Pd) | Remission  (none) | 1→0→0 |
| 14/F/30* | 104 | GTCS/Once, FS+/Daily | RS | Lt. medT T2 HSI | 0/102 | NMDAR | C | IVIG  +Steroid  (OxCBZ, LEV) | Remission | None | Remission  (LEV, ZNS) | 3→1→1 |
| 15/M/36 | 30 | FS+/Daily | RS | Lt. frontal swelling | 17/71 | NMDAR | C | Steroid  (VPA, TPM) | Remission | None | Remission  (VPA) | 3→1→1 |
| 16/M/33 | 21 | GTCS/Once, FS+/Daily | RS | Normal | 0/37 | NMDAR | C | Steroid  (VPA, DPH, TPM, LEV) | Remission | None  (Oral Pd) | Remission  (none) | 5→4→0 |
| 17/F/71† | 4 | GTCS/3 times | Normal | Normal | 0/67 | GABAb (+Hu) | C | IVIG  +Steroid  (LEV) | Remission | None | Remission  (LEV) | 5→4→4  (SCLC) |
| 18/M/62† | 62 | GTCS/3 times | GS | Normal | 11/39 | GABAb | P | IVIG  (TPM) | Remission | None | Remission  (TPM) | 5→3→2 |
| 19/F/53 | 53 | FBDS/Daily | RS | Normal | 2/99 | VGKC (LGI1) | P, M | Steroid  (LEV) | Remission | None | Remission  (None) | 1→1→0 |
| 20/F/24 | 24 | GTCS/Once | IED | Normal | 120/38 | NMDAR | P, M | Steroid  (LEV) | Remission | Plasmapheresis | Remission  (LEV) | 4→2→2 |
| 21/F/42 | 15 | FS+/Daily | IED | Bilateral medT T2 HSI | N/A | VGKC (LGI1) | C, P | IVIG  +Steroid  (VPA) | Remission | None  (Oral Pd) | >50% Reduction (LEV) | 3→2→1 |
| 22/F/42¶ | 58 | FBDS | IED | Bilateral medT T2 HSI | 0/33 | VGKC (LGI1) | C, M | Steroid  (LEV, OxCBZ, LTG, LZP) | >50% Reduction | IVIG  Plasmapheresis  RTXx7 | Remission  (LEV) | 3→3→2 |
| 23/F/70¶ | 3 | FBDS | IED | Bilateral medT T2 HSI | 2/30 | VGKC (LGI1) | C, P | IVIG  +Steroid  (LEV, OxCBZ) | >50% Reduction | RTXx 6  (Oral Pd) | Remission (LEV, TPM) | 3→2→0 |
| 24/F/18 | 14 | GTCS/twice | GS | Normal | 6/49 | NMDAR | M | IVIG  +Steroid  (LEV, TPM, CBZ) | >50% Reduction | RTXx7 | Remission (OxCBZ) | 5→5→4 |
| 25/F/24 | 17 | GTCS/Once, SE/Once | GS | Normal | 18/28 | NMDAR | C, P, M | IVIG  (OxCBZ, TPM) | >50% Reduction | Steroid  RTXx 4  (Oral Pd) | Remission  (LEV, TPM) | 5→5→4 |
| 26/F/37 | 11 | FS/Daily | IED | Normal | 15/22 | NMDAR | P, M | IVIG  +Steroid  (LEV, OxCBZ, CBZ) | >50% Reduction | RTXx 4  (Oral Pd) | Remission  (LEV, OxCBZ, VPA, ZNS) | 4→5→2 |
| 27/M/31 | 10 | FS+/Daily | GS | Normal | 385/73 | NMDAR | C, P | IVIG  +Steroid  (DPH, PB, TPM) | >50% Reduction | RTXx 1  (Oral Pd) | Remission  (LEV, TPM, CBZ) | 4→4→2 |
| 28/F/18 | 68 | FS+/Daily | GS | Normal | 5/39 | NMDAR |  | Steroid  (LEV, TPM, DPH, VPA, PGB) | >50% Reduction | None  (Oral Pd) | Remission | 2→1→1 |
| 29/F/39* | 364 | GTCS/ Daily | GS | Normal | 18/34 | NMDAR | P, M | Steroid  (LEV, TPM, DPH) | >50% Reduction | None | >50% Reduction  (CBZ) | 5→5→5 |
| 30/M/56 | 190 | FS+/daily | IED | Bilateral medT T2 HSI | 2/37 | VGKC (LGI1) | P | Steroid  (LEV, VPA, CBZ) | >50% Reduction | None  (Oral Pd) | >50% Reduction (LEV) | 2→1→1 |
| 31/F/74 | 74 | FS/Daily | Normal | Lt. frontal swelling | 165/235 | Yo | – | IVIG  +Steroid  (LEV, CBZ) | >50% Reduction | None | >50% Reduction  (LEV, LTG, PGB) | 2→2→1 |
| 32/F/20* | 107 | FS/Daily, SE/Once | IED | Normal | 3/31 | NMDAR | P | Steroid  (VPA, LTG, CBZ) | No change | None  (Oral Pd) | Remission  (LTG, DPH, LZP) | 5→3→0 |
| 33/M/57¶ | 45 | FBDS/Daily, GTCS/Once | RS | Lt. medT T2 HSI | 0/19 | VGKC (LGI1) | C, M | Steroid  (LEV, OxCBZ, CBZ) | No change | IVIG  RTXx 4 | Remission  (none) | 3→2→0 |
| 34/M/18 | 20 | FS+/Daily, SE/Once, | GS | Normal | 23/20 | NMDAR | P | Steroid  (LEV) | No change | IVIG  RTXx4 | Remission  (LEV, TPM, LCM) | 5→5→4 |
| 35/M/27 | 7 | GTCS/Weekly  SE/Once | RS | Normal | 61/41 | NMDAR | C, P | IVIG  +Steroid  (LCM, ZNS, PGB) | No change | RTXx8 | >50% Reduction  (LCM, ZNS, PGB) | 5→4→4 |
| 36/M/33* | 43 | FS+/Weekly, SE/Once | IED | Normal | 7/173 | Ma2/Ta | – | IVIG  (LEV, PB, CBZ) | No change | RTXx 6 | No change  (LEV, LCM, PGB) | 3→2→2 |
| 37/M/43 | 29 | FS/Daily | GS | Normal | 20/26 | NMDAR | C, P, M | IVIG  (VPA, DPH, TPM, .OxCBZ) | No change | RTXx8, | No change  (DPH, TPM) | 5→5→5 |
| 38/F/26 | 9 | FS+/Weekly, GTCS/3 times | RS | Bilateral medT swelling | 14/51 | NMDAR | C, P, M | IVIG  +Steroid  (LEV, PB) | No change | RTXx 2  (Oral Pd) | No change Deceased  (LEV, OxCBZ) | 5→5→6 |
| 39/M/72‡ | 72 | GTCS/Daily | GS | Normal | 34/47 | Ampiphysin | P | IVIG  +Steroid  (LEV, ZNS) | No change | None | No change Deceased  (LEV, ZNS) | 5→5→ 6 |
| 40/M/66† | 66 | GTCS/Daily | GS | Lt. medT T2 HSI | 0/104 | GABAb | P | Steroid  (LEV, TPM, VGB, LCM) | No change | IVIG | No change Deceased  (LEV, TPM, VGB, LCM) | 5→5→6 |
| 41/M/62 | 62 | GTCS/Daily | IED | Rt. Frontal HSI | 3/30 | VGKC (LGI1) | C | Steroid  (LEV, VPA, TPM) | No change | IVIG | No change Deceased  (LEV, TPM, DPH) | 4→5→6 |

Abbreviations: PTS, patients; N/A, not available; Age, age at seizure onset

Seizure types: FS, focal seizures without impaired awareness; FS+, focal seizures with impaired awareness; FBS, faciobrachial dystonic seizures; GTCS, generalized tonic–clonic seizures

Antiepileptic drugs: LEV, levetiracetam; TPM, topiramate; VPA, valproic acid; CBZ, carbamazepine; ZNS, zonisamide; VGB, vigabatrin; LCM, lacosamide; DPH, phenytoin; CNZ, clobazam; PB, phenobarbital; OxCBZ, oxcarbazempine; PGB, pregabalin; CZP, clonazepam

EEG, electroencephalography; IED, interictal epileptiform discharge; RS, regional slowing; GS, generalized slowing; medT, medial temporal; HSI, high signal intensity

Autoantibodies: LGI1, leucine-rich glioma inactivated 1; NMDA, *N*-methyl-d-aspartic acid receptor; Caspr2, contactin-associated protein-like 2; VGKC, voltage-gated potassium channel

Accompanying symptoms: C, cognitive impairment; P, psychiatric symptom; M, movement disorder

IVIG, intravenous immunoglobulin; Pd, prednisolone

RTX, rituximab; mRS, modified Rankin scale

*Patients 1, 4, 14, 29, 32, and 36 are also reported in reference #19. ¶Patients 2, 3, 9, 10, 12, 13, 22, 23, and 33 are also reported in reference #18.

†Patients 17 and 18 are also reported in reference #25. Patients 6, 7, and 8 are also reported in reference #26. ‡Patient 39 is also reported in reference #27.
