# Supplementary material for: Effect of Immunotherapy on Seizure Outcome in Patients with Autoimmune Encephalitis: A Prospective Observational Registry Study
Source: PLoS One. 2016 Jan 15;11(1):e0146455. doi: 10.1371/journal.pone.0146455 (PMC4714908; doi:10.1371/journal.pone.0146455)
Supplement: S2 Table — (DOC) [file pone.0146455.s003.doc]

S2 Table. Characteristics of patients with failure of the initial immunotherapy with or without rituximab

|  | Rituximab | No further Immunotherapy | *P*-value |
| --- | --- | --- | --- |
|  | n = 12 | n = 8 |  |
| Sex (male) | 6 | 4 | 1.00 |
| Age at seizure onset (years) | 32 (18–68) | 59 (18–74) | 0.157 |
| Seizure duration (days) | 15.5 (3–58) | 75.5 (22–385) | 0.001 |
| FBDS | 3 | 0 | 0.242 |
| SE | 4 | 1 | 0.603 |
| Abnormal MRI | 5 | 5 | 0.65 |
| EEG EDs | 5 | 4 | 1.00 |
| CSF leukocytosis | 9 | 4 | 0.356 |
| CSF protein elevation | 5 | 3 | 1.00 |
| Neural Ab type |  |  | 0.405 |
| NMDAR | 8 | 3 |  |
| VGKC | 3 | 2 |  |
| GABAb | 0 | 1 |  |
| Onconeuronal | 1 | 2 |  |
| Underlying malignancy | 0 | 2 | 0.123 |
| AED |  |  | 0.405 |
| Maximum No. of AED | 4 (2–6) | 3 (2–5) | 0.305 |
| AED escalation during the trial | 10 | 4 | 0.161 |
